# Primary results of abdominal aortic aneurysm screening in the at-risk residents in middle China

**DOI:** 10.1186/s12872-018-0793-5

**Published:** 2018-04-03

**Authors:** Kun Li, Kewei Zhang, Tianxiao Li, Shuiting Zhai

**Affiliations:** Department of Vascular and Endovascular Surgery, Henan Provincial People’s Hospital, Fuwai Central China Cardiovascular Hospital, NO.7 Weiwu Road, Zhengzhou, 450003 Henan People’s Republic of China

**Keywords:** AAA, Max-IAD, Prevalence rate, Ultrasound screening

## Abstract

**Background:**

There is a lack of information on the epidemiological data and risk factors associated with abdominal aortic aneurysm (AAA) in Chinese population. We reported the primary results from screening five-community population in Middle China for AAA.

**Methods:**

From March 2014 to October 2015, an AAA screening program was performed in three urban and two rural communities. These communities were randomly selected. All at-risk residents (a total of 6925) aged 40 years or older were invited to attend an ultrasound scan for AAA. At-risk population was defined as having a family history of first-degree relative diagnosed with AAA; or smoking and aged 55 years or older; or having a history of CAD, cerebrovascular disease, hypercholesterolemia, obesity (BMI ≧ 26 kg/m^2^) or hypertension.

**Results:**

The study investigated 5402 subjects and the mean age of them was 61.2 ± 10.4 years old. It included 2847 women aged 62.5 ± 10.4 years and 2555 men aged 59.7 ± 10.2 years. The mean maximum infrarenal aortic diameter (Max-IAD) was 15.0 ± 2.7 mm (from 4.1 to 51.5 mm). Eighteen people (aged 68.0 ± 10.4 years) with AAAs were detected (prevalence rate was 0.33%), and the prevalence rate in males was higher than in females (0.55% vs 0.14%, respectively, *P* = 0.009). Additionally, the screened subjects aged 55 to 75 years had a higher prevalence rate of AAA than other age groups (0.51% vs 0.11%, respectively, *P* = 0.016).

**Conclusion:**

The mean Max-IAD of the screened population in Middle China was apparently small by comparison with other reports. The result of low prevalence rate of AAA didn’t support routine screening in Chinese population. The at-risk males aged 55 to 75 years should be targeted for further screening.

## Background

Abdominal aortic aneurysm (AAA) is usually undiagnosed due to no symptoms before rupture. Its mortality rate is high even if the operation time is allowed [1–3]. A population-based ultrasonography screening program has been advocated because screening for AAA has been shown to reduce AAA-related mortality and provide an opportunity to modify cardiovascular risk and improve the general health of the screening population [4]. Several randomized controlled trials suggested that early detection of AAA by ultrasound screening could significantly reduce all-cause mortality in men aged ≥65 years and the cost of treatment for AAA during long-term follow-up [5–8].

Considering the balance between cost-effectiveness and incidence, high-risk population shall be targeted for screening trial. It has been proved that the increased risk of AAA includes advanced age, a history of smoking, atherosclerotic burden, and a family history of aneurysmal disease [7, 9]. Several reports demonstrated that Asians had a lower prevalence rate of AAA than that was reported in Caucasians after adjusting for all other known influencing factors [10–14]. However, there is a lack of information on the epidemiological data and risk factors of AAA disease in Chinese mainland. Consequently, a community-based screening program for AAA was performed in residents of randomly selected urban and rural communities in Middle China because of the highest uptake. The aim of this study was to report the primary results of the screened population for the prevalence rate of AAA and associated risk factors with AAA in Chinese population.

## Methods

From March 2014 to October 2015, an AAA screening program was performed in five communities. Five communities (including three urban and two rural communities) were randomly selected from Zhengzhou city in Middle China. The geographic areas of our screening program contain a significant difference between urban and rural areas because of the different socioeconomic status, the dietary and lifestyle habits. The total population aged 40 years or older was estimated at 12,550 based on demographic statistics. A population-based sample of subjects aged 40 years or older was enrolled and invited to complete a risk-factor questionnaire in screening sites or by telephone before screening.

The at-risk population was enrolled according to the assessment of risk-factor questionnaires which included demographics, residential area, weight, height, self-reported smoking, self-reported drinking history (alcohol consumption, contained years of drinking), hypertension or diabetes (as a history of this disease treated through diet or medication), hyperlipidemia, coronary artery disease (CAD, as a history of myocardial infarction), angina pectoris of coronary revascularization, cerebrovascular disease (as a history of previous transient ischemic attack or stroke), and family history (first-degree relative diagnosed with AAA). The weight and height were used to calculate body mass index (BMI). Self-reported smoking was defined as a lifetime used 100 cigarettes and contained information of daily cigarette consumption (packs/day) in addition to years of smoking. Twelve thousand and one hundred questionnaires were collected for further risk assessment. At-risk population was defined as having a family history of first-degree relative diagnosed with AAA, smoking population aged 55 years or older, or having a history of CAD, cerebrovascular disease, hypercholesterolemia, obesity (BMI ≧ 26 kg/m^2^) or hypertension. The final cohort included 5402 screened subjects (78%) in the 6925 at-risk eligible subjects.

Before ultrasound scan, all screened individuals had received a physical examination including blood pressure, weight and height. The systolic blood pressure and diastolic blood pressure were measured in both upper extremities. When blood pressure in both upper extremities measurements inconsistent, the higher one was used. The blood test was carried out including fasting blood-glucose, triglyceride, serum total cholesterol (TC), low density lipoproteins (LDL), high density lipoprotein (HDL), and homocysteine level.

All screened individuals underwent an ultrasound screening of AAA to identify the maximum anteroposterior diameter of the infrarenal aorta according to the leading edge principle in longitudinal section of aorta [15]. Ultrasonic examination was performed by an ultrasound machine (SonoSite 180 PLUS, USA) with a 3.5 MHz transducer. AAA was identified as a maximum infrarenal aortic diameter (Max-IAD) exceeded 30 mm or had 50% increase or more in diameter compared with the mean normal. The data was entered into a specific database.

### Statistical analysis

SPSS 19.0 software package was used for the statistical analyses in this study. Student *t* test or one-way analysis of variance (ANOVA) was used to compare quantitative variables. The difference was statistically significant (*P* < 0.05).

## Results

### Characteristics of the enrolled population

**Fig. 1:**
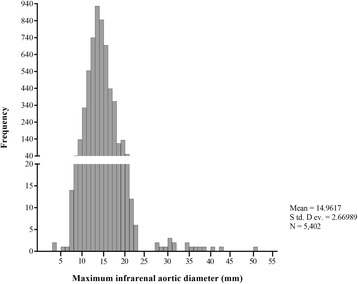
The distribution of maximum infrarenal aortic diameters for the entire screened cohort (including a selective histogram of the size distribution of infrarenal aortic diameters > 28 mm)

**Fig. 2:**
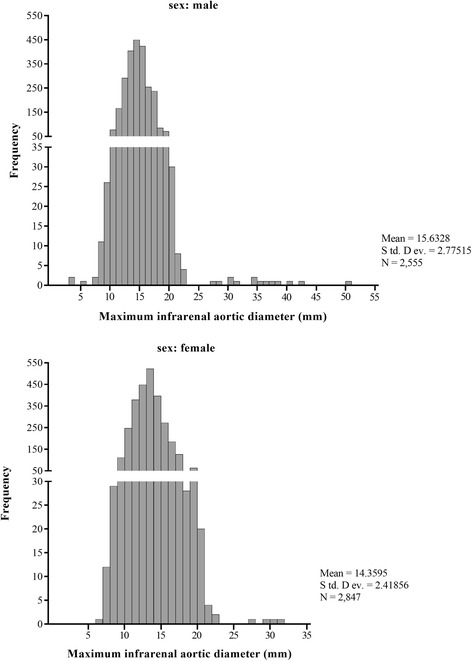
The distribution of maximum infrarenal aortic diameters for the male and female at-risk individuals

The final at-risk cohort contained 5402 screened subjects aged 61.2 ± 10.4, including 1682 subjects (31.13%) under 55 years old, 3166 subjects (58.61%) aged 55 to 75, and 554 subjects (10.26%) over 75 years old. As shown in Table 1, the urban residents accounted for 48.17% (*n* = 2602). Moreover, 1481 subjects (27.42%) had a history of stroke, 983 subjects had CAD (18.20%), 1681 subjects (31.12%) had a history of diabetes, 3685 subjects (68.22%) had hypertension, 1275 subjects (23.60%) had self-reported smoking, and 1142 subjects (21.14%) had drinking history in total individuals. The smoking rate for men was higher than that for women (*P* < 0.001, Table 1), and approximately 30% cohort were obese or overweight (BMI ≧ 26 kg/m^2^). Additionally, the mean Max-IAD of the screened population was 15.0 ± 2.7 mm (range from 4.1 to 51.5 mm, Figs. 1 and 2). The mean Max-IADs of different age groups were 14.9 ± 2.3 mm (under 55 years), 15.1 ± 2.7 mm (55–65 years), 15.0 ± 3.0 mm (65–75 years) and 14.3 ± 2.7 mm (over 75 years), respectively. Furthermore, the Max-IAD in females was smaller than in males (14.4 ± 2.4 mm vs 15.6 ± 2.8 mm, *P* = 0.001, Table 1). Both the mean and median Max-IAD of the total population were 15 mm (95% CI, 14.9 to 15.0 mm, Fig. 2).

**Table 1:** Characteristics of screened at-risk population by gender

Characteristics	Overall	Male	Female	*P*
Screened individuals, %	5402 (100)	2555 (47.30)	2847 (52.70)	0.615
Age				0.009*
Overall age	61.2 ± 10.4	59.7 ± 10.2	62.5 ± 10.4	
Age without AAA^a^	61.2 ± 10.4	59.6 ± 10.2	62.5 ± 10.3	
Age with AAA	68.0 ± 10.4	57.1 ± 10.5	71.3 ± 11.2	
Screening areas				0.041*
Urban,%	2602 (48.17)	1216 (22.51)	1386 (25.66)	
Rural,%	2800 (51.83)	1339 (24.79)	1461 (27.05)	
History of stroke,%	1481 (27.42)	690 (12.77)	791 (14.64)	0.063
CAD, %^b^	983 (18.20)	368 (6.81)	615 (11.38)	0.004*
Diabetes,%	1681 (31.12)	779 (14.42)	902 (16.70)	0.635
Hypertension, %	3685 (68.22)	1688 (31.25)	1997 (36.97)	0.046*
Smoke(years), 95% CI	1275 (23.60)	1237 (22.90)	38 (0.70)	0.000*
Overall max-IAD (mm)^c^	15.0 ± 2.7	15.6 ± 2.8	14.4 ± 2.4	0.001*

^a^abdominal aortic aneurysm; ^b^coronary artery disease; ^c^The maximum infrarenal aortic diameter; *P < 0.05

### Epidemiological data and risk factors of AAA

Eighteen AAAs were detected during the study, with total prevalence rate of 0.33% in the eligible screened individuals. Clinical characteristics of AAAs were shown in Tables 2 and 3. The prevalence rate of AAA in male was higher than in female (0.55% vs 0.14%, *P* = 0.009). The difference in mean age between men (57.1 ± 10.5 years) and women (71.3 ± 11.2 years) was statistically significant (*P* = 0.001). Additionally, the prevalence rate of AAA had significant differences in different age groups, and results showed a higher AAA prevalence in age 55–75 years than that in the other age groups (0.51% vs 0.11%, *P* = 0.016). The prevalence rate of AAA in the at-risk urban population was higher than that in the at-risk rural population (0.50% vs 0.18%, *P* = 0.041). Moreover, the diameter of aneurysms ranged from 28 to 51.5 mm (mean Max-IAD was 35.1 ± 6.0 mm). Above 80% of all screening-detected AAAs had over 40 mm in aneurysms diameter, and the size distribution of Max-IAD over 28 mm (Fig. 1).

**Table 2:** Characteristics of the screening at-risk cohort with and without abdominal aortic aneurysm (AAA) by age, gender, screening areas and medical history

variables	Without AAA (*n* = 5384)	With AAA (*n* = 18)	*P*
Sex			
Male,%	2541 (47.20)	14 (77.78)	0.009*
Female,%	2843 (52.80)	4 (22.22)	0.009*
Age group			
≤ 55 yrs.,%	1682 (31.24)	0	
> 55, ≤65 yrs., %	1940 (36.03)	8 (44.44)	0.016*
> 65, ≤75 yrs., %	1210 (22.47)	8 (44.44)	0.016*
> 75 yrs	552 (10.25)	2 (11.11)	0.016*
Screening areas			
Urban,%	2589 (48.09)	13 (72.22)	0.041*
Rural,%	2795 (51.91)	5 (27.78)	0.041*
Medical history			
CAD,%^a^	975 (18.11)	8 (44.44)	0.004*
Diabetes,%	1677 (31.15)	4 (22.22)	0.061
Hypertension,%	3668 (68.13)	17 (94.44)	0.001*
History of stroke,%	1470 (27.30)	11 (61.11)	0.001*
Family history of AAA, %^b^	2 (0.04)	3 (16.67)	0.000*

^a^coronary artery disease; ^b^abdominal aortic aneurysm; *P < 0.05

**Table 3:** Characteristics of the screening at-risk cohort with and without abdominal aortic aneurysm (AAA) by risk factors

Risk factor	Without AAA^g^(n = 5384) 95% CI	With AAA (n = 18) 95% CI	*P*
Mean age	61.15 (60.87–61.43)	68.00 (62.81–73.19)	0.005*
Hypertension (years)	5.55 (5.34–5.77)	7.22 (4.13–10.31)	0.379
Smoke (years)	6.43 (6.08–6.78)	21.28 (11.09–31.47)	0.000*
Drinking history (years)	5.62 (5.30–5.94)	16.39 (5.57–22.20)	0.000*
BMI (kg/m^2^)	32.11 (24.29–39.93)	25.58 (23.51–27.65)	0.925
SBP (mmHg)^a^	141.46 (140.95–141.98)	142.89 (134.81–150.97)	0.753
DBP (mmHg)^b^	88.93 (88.63–89.23)	87.94 (83.78–92.11)	0.713
FBG (mmol/L)^c^	6.65 (6.59–6.70)	6.79 (5.61–7.97)	0.770
Triglyceride (mmol/L)I	2.26 (2.20–2.33)	1.99 (0.94–3.03)	0.630
TC (mmol/L)^d^	5.18 (5.13–5.23)	6.58 (2.33–10.82)	0.003*
LDL (mmol/L)^e^	2.91 (2.89–2.93)	2.47 (2.15–2.78)	0.028*
HDL (mmol/L)^f^	1.40 (1.38–1.42)	1.42 (1.19–1.65)	0.929
Homocysteine (μmol/L)	18.96 (18.58–19.33)	19.89 (16.00–23.77)	0.780

^a^systolic blood pressure; ^b^diastolic blood pressure; ^c^fasting blood-glucose; ^d^serum total cholesterol; ^e^low density lipoproteins; ^f^high density lipoprotein; ^g^abdominal aortic aneurysm; * *P* < 0.05

The risk factor distribution was displayed in Tables 2 and 3. AAAs with the old-age, history of stroke, hypertension, CAD, TC, LDL, positive family history of AAA, smoking and drinking history had higher morbidity than AAAs without these risk factors (*P* < 0.05). Additionally, according to the results of multivariable regression analysis for predictors of AAA, history of stroke, CAD, smoking history, positive family history and TC value were independently associated with AAA (Table 4).

**Table 4:** Results of multivariable regression analysis for predictors of abdominal aortic aneurysm (AAA)

Risk factor	Odds ratio	95% CI	*P*
History of stroke	0.244	0.088–0.677	0.007
CAD^a^	0.263	0.095-0.728	0.010
Diabetes	1.182	0.787–1.774	0.420
Family history of AAA^b^	1.3	1.1–1.4	0.015
Hypertension	1.006	0.953–1.062	0.831
Smoking	1.049	1.018–1.081	0.002
Drinking history	1.017	0.985–1.049	0.306
BMI	0.934	0.814–1.072	0.332
SBP^c^	1.006	0.977–1.035	0.693
DBP^d^	1.010	0.961–1.062	0.686
FBG^e^	1.101	0.898–1.350	0.354
Triglyceride	0.969	0.745–1.262	0.818
TC^f^	1.092	1.025–1.164	0.006
LDL^g^	0.493	0.251–0.968	0.040
HDL^h^	1.082	0.592–1.977	0.799
Homocysteine	1.000	0.967–1.034	0.999

^a^coronary artery disease; ^b^abdominal aortic aneurysm; ^c^systolic blood pressure; ^d^diastolic blood pressure; ^e^fasting blood-glucose; ^f^serum total cholesterol; ^g^low density lipoproteins; ^h^high density lipoprotein

## Discussion

AAA is a progressive disease with increasing age. The high prevalence of AAA in Caucasian population is well reported [10, 16]. Early detection of AAA can save life, and offer a much safe and effective treatment to emergency intervention of AAA rupture patients [2]. However, the screening target population is different according to the different recommendations [17–19]. The Society for Vascular Surgery recommended an AAA screening for women aged 65 years or older who have smoked or have a family history of AAA and men aged 55 years or older who have a family history of AAA [18]. Despite current studies recommend screening for high-risk groups, it has been also debated that who should be screened and which risk factors can potentially be used to confirm high-risk population for cost-effective of screening [20–22]. A previous study found that about half of the patients with AAA were not screened according to the current guidelines [8]. Moreover, it is also doubtful that the results of the target-population screening reported by Western studies are suitable for Chinese screening for AAA. Consequently, our present study expanded the screening target population. We screened subjects aged over 40 years who have well-known risk factors related to AAA, aiming to explore detailed information on epidemiological data of AAA in Chinese population.

There are few studies on accurate prevalence rate of AAA in Chinese population. It has been reported that the mean Max-IAD was about 15 mm, and prevalence rate of AAA was 0.11% in the Chinese hypertensive adults, which showed smaller mean abdominal aortic size and lower prevalence of AAA in Chinese population than in Western population [23]. A previous study also showed that the mean Max-IAD in China was 17.5 mm for men and 14.8 mm for women [13]. Therefore, a diagnosis of AAA with Max-IAD of > 30 mm in China may be questioned. Our results showed small abdominal aortic size of the mean Max-IAD was 15.0 ± 2.7 mm in the overall screened population. According to the recommendation of the Society of Vascular Surgery and International Society for Cardiovascular Surgery, we defined AAA as an Max-IAD of > 30 mm or having 50% increase or more in diameter compared with the mean normal [24]. We found that the prevalence rate of AAA was low and only 18 AAAs were found (0.33%). The result was similar to the previous study [14, 23]. Based on our present study, there was a lower prevalence rate of AAA when its definition as Max-IAD of > 30 mm, only 18 AAA patients (AD> 30 mm) in our screened individuals.

Future screening strategies for AAA should be targeted those high-risk individuals. The United States Preventive Services Task Force (USPSTF) commanded that risk factors for AAA included older age, male sex, smoking history, positive family history of aneurysms, hypertension, cerebrovascular disease, coronary artery disease, atherosclerosis, hypercholesterolemia and obesity [17]. Our results showed that the prevalence rate of AAA was significantly higher in men than in women, and male was older (71.3 ± 11.2 years) than female (57.1 ± 10.5 years) in the total AAAs, which were consistent with previously reported results [10, 14]. The screened individuals aged 55–75 years had a higher AAA prevalence rate than other age groups. Our result confirmed this observation that the at-risk male aged 55–75 years should be targeted for further screening because of the high prevalence rate of AAA.

The geographic areas of our screening program contained a significant difference between urban and rural areas with different dietary and lifestyle habits. The prevalence rate of AAA in the urban population was higher than in the rural population, which might be due to the higher consumption of fast food, meat and lack of exercise in urban population. This result reinforced the role of lifestyle in forming AAA [8]. Meanwhile, the result of our study reaffirmed that smoking, drinking history, positive family history of AAA, history of stroke, CAD, hypertension and hyperlipidemia were more likely to increase the prevalence of AAA. We found that history of stroke, CAD, smoking history, TC value and positive family history were independently associated with AAA. It has been proved that smoking as the major environmental risk factor has a strong positive association with AAA, which can increase the prevalence and the risk of rupture of AAA [8]. Many patients with AAA often have other atherosclerotic vascular diseases, which is the same as our patients, and these diseases have common risk factors [8]. It is also known that hyperlipidemia is involved in the formation of vascular wall atherosclerosis and AAA. Atherosclerotic vascular diseases and AAA have been traditionally regarded as two separate angiosis but have some common pathogenetic backgrounds [1]. Previous studies indicated that high level of homocysteine played a role in the pathogenesis of atherosclerotic vascular disease [25]. The present study found that plasma homocysteine level was not directly associated with AAA, which indicated that atherosclerosis and AAA had different pathogenesis.

However, this study had several limitations. Our study was composed of a highly selected group of subjects with one or several risk factors of arteriosclerotic diseases. The present screening trial was mainly only focused on the residents of several local communities, and only 18 AAAs were found due to the low prevalence of AAA in Middle China. Meanwhile, because of lacking long-term follow-up, our study was a cross-sectional study, and the long-term benefit of screening was uncertain. Therefore, the large-scale screening study is indispensable to validate our finding.

## Conclusions

This was the first report on the large screening program for AAA in residents of the urban and rural communities in Middle China. This study provided a good evidence that the mean Max-IAD in Chinese population was apparently small by comparison with other reports. The result of significantly low prevalence rate of AAA in highly selected at-risk group did not support routine screening in Chinese population. The male aged 55–75 years should be targeted for further screening because of the high prevalence rate of AAA. The results of our study also confirmed that smoking, drinking history, atherosclerotic burden, hypertension, hyperlipidemia and positive family history of AAA were more likely to increase the prevalence rate of AAA.

## Abbreviations

AAA: Abdominal aortic aneurysm
BMI: Body mass index
CAD: Coronary artery disease
HDL: High density lipoprotein
LDL: Low density lipoproteins
Max-IAD: Maximum infrarenal aortic diameter
TC: Total cholesterol
USPSTF: The United States Preventive Services Task Force
